# The p.(Gly111Arg) *ABCC8* Variant: A Founder Mutation Causing Congenital Hyperinsulinism in the Indian Agarwal Community

**DOI:** 10.1111/cge.14657

**Published:** 2024-11-27

**Authors:** Vandana Jain, Venkatesan Radha, Viswanathan Mohan, Matthew N. Wakeling, Jasmin J. Bennett, Sarah E. Flanagan

**Affiliations:** ^1^ Division of Pediatric Endocrinology, Department of Pediatrics All India Institute of Medical Sciences Delhi India; ^2^ Department of Molecular Genetics Madras Diabetes Research Foundation (MDRF) Chennai Tamil Nadu India; ^3^ Department of Diabetology MDRF & Dr. Mohan's Diabetes Specialities Centre Chennai India; ^4^ Department of Clinical and Biomedical Science University of Exeter Exeter UK

**Keywords:** CHI, founder variant, hyperinsulinism, India

## Abstract

Loss‐of‐function *ABCC8* variants are the commonest cause of congenital hyperinsulinism. On a systematic search of our databases, the p.(Gly111Arg) *ABCC8* variant was identified in 26 individuals, of which 23 were from the Indian Agarwal community. Haplotype analysis subsequently confirmed that p.(Gly111Arg) is a founder variant in the Agarwal population.

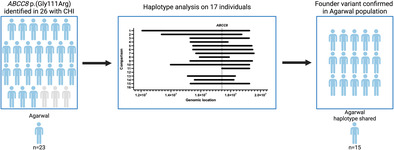

Congenital hyperinsulinism (CHI) usually presents in the newborn period as persistent hypoglycemia due to inappropriate release of insulin from β‐cells. Most affected newborns require prolonged hospital stay and are at risk of neurodevelopmental sequelae [1]. The commonest genetic cause of CHI is loss‐of‐function variants in the *ABCC8* gene, encoding the SUR1 subunit of the pancreatic K‐ATP channel [1].

*ABCC8* is highly polymorphic with founder variants described in the Irish, Finnish, Ashkenazi Jewish, Bedouin, Spanish, and Hispanic populations [2, 3]. In 2013, the p.(Gly111Arg) *ABCC8* variant was flagged as a possible founder variant in the Indian population [4]. Subsequently, we observed that this variant contributed disproportionately to the CHI in individuals treated at our center (AIIMS, Delhi), and was present only in patients from the Agarwal community.

To investigate this further we searched for individuals with CHI and an *ABCC8* p.(Gly111Arg) variant who had presented to AIIMS Delhi within the last 10 years, or had been referred for genetic testing to the Exeter Genomics Laboratory or the Madras Diabetes Research Foundation (MDRF).

We identified 26 individuals with a p.(Gly111Arg) variant (*n* = 9 AIIMS Delhi, *n* = 15 Exeter, and *n* = 2 MDRF). Twenty‐three children were identified as Agarwals based on their declared community or family name, one patient from the UK, had a surname which translated to “Indian” in Hindi, suggesting north‐Indian ancestry; one patient each was Vietnamese and Iranian. The variant was homozygous in 11 individuals, in trans with a second pathogenic *ABCC8* variant in 9, and paternally‐inherited (consistent with focal disease) in six.

Samples from 17 heterozygous parents underwent SNP array analysis (Illumina GSA Arrays “Infinium iSelect 24x1 HTS Custom Beadchip Kit”), which identified a haplotype shared by the 14 parents from the Agarwal community and the one parent with north Indian ancestry, but not the two parents from Vietnam or Iran (Figure 1). Whilst a recombination event within a small region containing *ABCC8* could not be excluded, the ethnicity/nationality of these individuals together with descriptions of the p.(Gly111Arg) in non‐Indian cohorts suggests it has arisen on multiple alleles over time in different populations.

**FIGURE 1:**
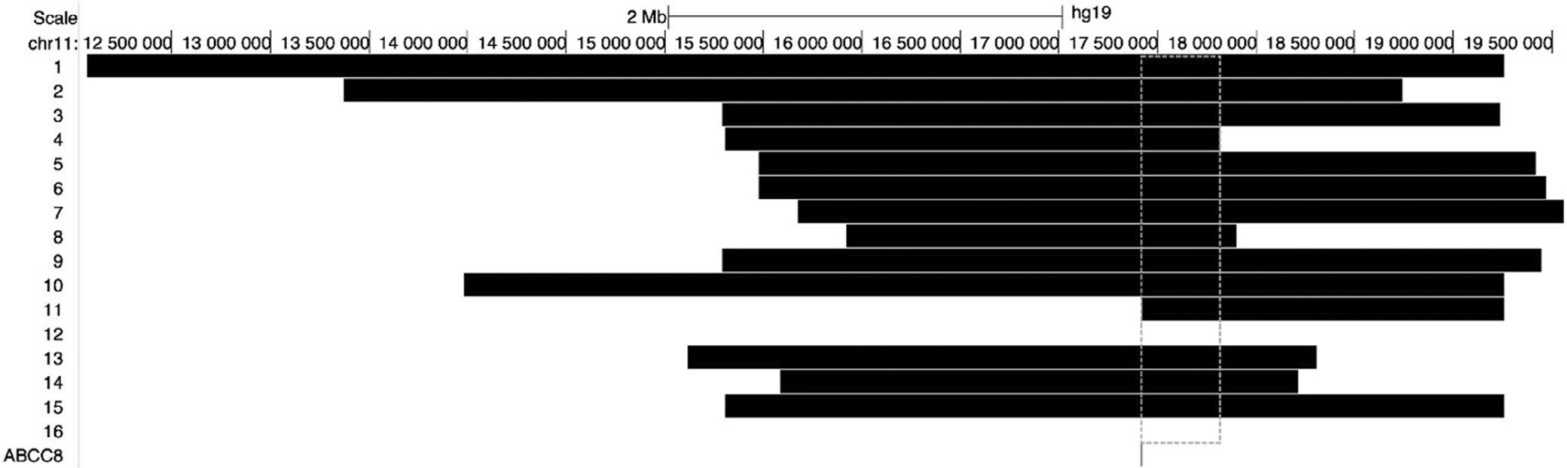
Haplotype results for individuals with a p.(Gly111Arg) c.331G>A *ABCC8* variant. Regions containing ≥ 150 consecutive variants over the depicted *ABCC8* gene were called in each individual. Data from each individual was compared against one individual known to be part of the Agarwal community. Each numbered line indicates the region of shared genetic variants for each comparison. Comparisons of individual's numbered 12 (Iranian) and 16 (Vietnamese) with an individual from the Agarwal community did not identify a shared haplotype. The hashed line indicates the minimal shared region in 15 individuals (GRCh37:Chr11:17 421 790–17 816 190).

In 5 of the 8 (62.5%) individuals from the Agarwal community with compound heterozygous variants, the second variant was p.(Arg74Gln) (c.221G>A) suggesting that this too may be a founder variant. Further analysis will be needed in corroborating this observation.

Agarwals are a large community predominantly residing in the northern, central and western regions of India and are estimated to account for 0.7%–1% of the Indian population [5]. There are 18 clans (gotras) in this community and marriages typically follow the convention of clan exogamy (only taking in the paternal side of the family), and caste endogamy. This practice has led to genetic homogeneity within the community with many founder variants for rare disorders described [5].

This study confirms that p.(Gly111Arg) is a founder variant in the Agarwal community. These results will enable continued monitoring of the variant, allow for rapid prenatal screening for CHI, as well as quick molecular diagnosis in babies with CHI in this community.

## Author Contributions

V.J. conceived the study, collated AIIMS data, and drafted the manuscript. V.R., V.M., and J.J.B. collated genetic data. M.N.W. performed haplotype analysis. S.E.F. interpreted genetic data, co‐drafted the manuscript, and acts as the guarantor.

## Ethics Statement

Informed consent was obtained from all patients/parents. Ethical approval was obtained from AIIMS Delhi (IEC‐109/5.2.21, RP‐26/2021) and Genetic Βeta‐Cell Research Bank (517/WA/0327).

## Conflicts of Interest

The authors declare no conflicts of interest.

### Peer Review

The peer review history for this article is available at https://www.webofscience.com/api/gateway/wos/peer‐review/10.1111/cge.14657.

## Data Availability

Restrictions apply to the availability of some data generated or analyzed during this study to preserve patient confidentiality. Access to the genetic data is available only through collaboration to experienced teams working on approved studies examining the mechanisms, cause, diagnosis and treatment of diabetes and other beta cell disorders. Requests for collaboration will be considered by a steering committee following an application to the Genetic Beta Cell Research Bank (https://www.diabetesgenes.org/current‐research/genetic‐beta‐cell‐research‐bank/).
